# Dental Education

**Published:** 1859-08

**Authors:** J. Taft


### DENTAL REGISTER OF THE WEST.

Vol. XIII.] AUGUST, 1859. [No. 2.

### Original Essays and Communcations.

### DENTAL EDUCATION.

BY J. TAFT.

The basis of a good dental education, is a good general *literary* and *scientific* education; without this, comparatively little can be accomplished in any professional study. There are two considerations worthy of notice here: in order to pursue most efficiently any professional course, the mind should be thoroughly trained, and habituated to thorough investigation, and close reasoning. The mind should have such a prior course of training, as would enable it to comprehend, and act upon those subjects pertaining to professional study, with the least possible bias, and liability to err. In a literary education, the student may embrace wrong opinions, and yet no injury result to him therefrom; not so however in regard to scientific, and still less so in regard to professional education. An error in professional education is a matter of grave moment. There will be outcroppings from it, all along through the professional life, of one who has thus imbibed wrong notions; he will thus be liable to be led astray at important points—thrown off the track, upon which he must, at cost, right himself, or lie in chaotic ruins. The errors that a man may commit in a literary education, will for the most part be corrected, as he passes along through an active life. There is, for such an one, far more correcting agencies, than for the man of science or profession. Literary attainments are also valuable, in elaborating, and bringing more fully to view, the principles involved in science. Literary training should always be thorough whatever be its range. In extent it should be by no means circumscribed. There is almost universally, a greater deficiency in thoroughness than extent. It is not all important, that the dental student should be a Greek and Hebrew scholar, nor, in every sense, a thorough mathematician, though it would be better if he were, yet it *is* all important that he should be perfectly familiar with the literature of his own language, for this is to be one of the means of his professional attainments. At how great a disadvantage does the man labor in professional studies, who is ignorant of the derivation and definition of much of the language he is compelled to use. In order to make progress, the definition of every word should be clearly understood, so that every idea presented to the mind of the student, should be closely apprehended, at the first presentation ; the simple comprehension of a subject will by no means satisfy the true student; but the proposition or principles set forth, should find a verification, by his own reasoning, or by such facts as he may be able to call to his aid, which will in many instances make positive demonstrations. It is always humiliating to see a man receiving principles or theories, simply upon the declaration of another, and yet the ignorant man finds himself exactly in this position, he receives the great proportion of his ideas upon the mere simple assertion of others. The illiterate man is unable to comprehend the truths of science, because of the opacity of the medium through which they must pass to him, and from the inability of his untrained mind properly to receive, and efficiently to act on them. In these respects, and others that might be named, doos the untrained mind labor at a disadvantage, while engaged in scientific studies.

In any professional study, a thorough knowledge of the common sciences is almost indispensable. They may all be made auxiliary to any profession, and to the dental profession quite as much so as to any other. The study of science trains the mind, but more than this, it gives an acquaintance and familiarity with an extensive range of subjects, any one of which may be called to aid, when occasion requires; but how is this to be done by the student of a profession, unless he first makes the subject his own. It is not necessarily true, that when we become scientific, we are less practical, but the very opposite is true, under proper culture; the more scientific a man is, the more practical he becomes.

The man of science has maps, and charts, and compass, to aid him, even though he should enter upon an unexplored field; but especially when he assays to go, where others have gone before him, does the way become easy, and he is almost without effort borne along. By the aid of general science, the study of a speciality becomes easy and even a delight, but without, the way is dark, uncertain and laborious. Science is a light to professional men, all the way through.

In thus speaking of scientific and literary attainments, of course it is only the real attainments that are referred to, and not the superficial and ephemeral education possessed by so many—the mere routine knowledge obtained by "*going through the books.*" In almost all cases such an education, in any department, is worse than nothing at all, it certainly is, unless the possessor is aware of the fact and does not rely upon it, more than it will warrant. When a man has superficial attainments, and is disposed to rely upon them, he will find his course a rough and a hard one. The man of great attainments is always a great worker.

Other professions have set standards of literary and scientific attainments for those who would become their pupils.—This has been wholly disregarded by the dental profession, owing probably to a want of efficient organization in its ranks.

All who have taken a fancy to it, have entered it regardless of elementary training.

We are admonished by one cautious friend, that this is rather a delicate subject. Well, delicate or not, we have felt an hundred times like handling it without gloves. What! our profession cramped, throttled and stultified, and yet no one dare to speak of one of the great causes of the difficulty; shall our colleges receive students who cannot write a sentence of the English language correctly? such at least ought not to be the case.

I would suggest that our dental colleges, by a uniform course of action on this subject, could accomplish much; let them fix some point to which they will rigidly adhere. It may be best to fix the standard low, but the applicant should at least be able to speak and write his native language correctly, and understand the elements of some of the common useful sciences. There are many reasons why he who seeks to enter the dental profession, should possess literary and scientific attainments of a high order. First, the profession is in a stage of development, and it requires the proper kind of nourishment—men who are willing to work, with a view to the upbuilding and strengthening of a young profession—men with clear heads, willing hearts, and strong hands.

Again, there is a great diversity of subjects pertaining to dental study. Art and science both enter into it. The dentist must be both an artist and a scientifician. He should give attention to all the branches of medical education. He has also to study mechanics, and many of the manipulations pertaining thereto. He should also study the symmetry and beauty of the human face, in all its phases. Some attainments are also neccssary in order that the dentist may occupy a respectable position in his profession, and with other professions. Until within comparatively recent date, there was no recognition of the dentist, by any body, above that of a common tradesman or artizan. It is now, however, beginning to be otherwise; there are some men in the dental professsion, whose attainments and abilities, not only in their speciality, but in science generally, compels a recognition, both by the learned and the unlearned. There has been such a mass of ignorance in our profession, that the better part could not carry it along, but had to sink, for the time, with it; but there is a sloughing off, a separation being made, so that the good will more rapidly rise, and the bad with more velocity sink.

In the absence of true dental education, we generally find *assumption*, *arrogance*, *empyracism*, *incompetency*, and decadence in public estimation.

And beyond all the attainments referred to, the profession needs men of sterling integrity, high honor and moral honesty; men who have the skill and honesty to make good operations; and the integrity and firmness to charge what they are worth; men who are willing to meet and determined to conquer difficulties. All such will be received with open arms into the ranks of the dental profession.

